# Transparent, Conductive and Hydrophobic Cellulose Paper Fabricated by Photopolymerization

**DOI:** 10.3390/molecules31203711

**Published:** 2026-10-16

**Authors:** Ruiping Tong, Huan Ye, Haojie Ren, Longjun Su, Junfei Xu, Dandan Fang, Jiayi Li, Tengfei Li

**Affiliations:** Zhejiang Key Laboratory of Intelligent Manufacturing for Aerodynamic Equipment, College of Mechanical Engineering, Quzhou University, Quzhou 324000, China; tongpinp@qzc.edu.cn (R.T.); yeahhuan1024@163.com (H.Y.); jayren2000@163.com (H.R.); dyxslj12101007@163.com (L.S.); fangdd2004@163.com (D.F.); hpxhzgsj@163.com (J.L.)

**Keywords:** hydrophobic cellulose paper, photopolymerization, transparency, ionic conductivity, flexible optoelectronic devices

## Abstract

Transparent cellulose papers used as substrates have gained increasing attention in the flexible optoelectronics field. However, significant challenges remain in overcoming the hydrophilicity of transparent cellulose papers and endowing them with conductivity for multifunctional applications. Herein, a new avenue for the construction of highly transparent, ionically conductive, and hydrophobic cellulose paper (HCP) via photopolymerization is reported. The photopolymerization of ionic liquid-containing photopolymerizable precursor and allyl cellulose on the surface of allyl cellulose paper yields robust functional surfaces. The obtained HCP displays high transparency (>90% in a wide wavelength range), better surface hydrophobicity (water contact angle ~127°), and high thermostability (onset thermal decomposition ~220 °C). In addition, the HCP exhibits reliable, sensitive and reproducible abilities to external stimulus. Significantly, the ionic conductive HCP has successfully been used for the construction of integrated and flexible optoelectronic devices. This promising strategy provides new insights into tailoring the surface hydrophobicity and functionality of cellulose paper for flexible electronic devices.

## 1. Introduction

Transparent cellulose papers used as substrates have been widely investigated in the flexible optoelectronics field due to their significant advantages, such as having good flexibility and high transparency and being environmentally friendly and renewable [1,2,3,4]. Significantly, the transparent cellulose paper is susceptible to moisture and water due to abundant hydrophilic hydroxyl groups in cellulose molecular chains, which would reduce the shape stability of the transparent cellulose paper, resulting in unstable electrochemical properties of paper-based flexible optoelectronic devices [5,6,7]. Therefore, several methods have been proposed to prepare transparent and hydrophobic cellulose paper, which mainly include chemical modifications of hydroxyl groups (e.g., acetylation [8], silylation [9], and polymer grafting [10]), and the use of hydrophobic coatings (e.g., fluorinated nanoparticles [11] and low surface energy materials [12]) combined with transparent cellulose paper via surface spraying or impregnation. Despite their impressive hydrophobic performance, the above strategies either endow cellulose paper with only a single hydrophobic property, which limits their application in multifunctional environments, or they struggle to balance overall performance, e.g., the utilization of hydrophobic coating usually leads to decreased transparency, and weak interfacial interaction between cellulose paper and hydrophobic coating results in poor stability [13,14].

In addition, electrical properties are a necessary prerequisite for the normal operation of flexible optoelectronic devices. Therefore, intensive efforts, generally utilizing electronically conductive materials (e.g., conductive polymers [15,16], metal-based nanowires [17,18], and carbon-based materials [19,20]) and ionically conductive materials (e.g., deep eutectic solvents [21] and solvated salts [3]), have been invested in developing transparent and conductive cellulose paper. Noticeably, resultant conductive cellulose paper prepared from electronic conductive materials generally suffers from decreased transparency and/or abrupt hard-soft interfaces [21,22]. In contrast, ionic conductive materials can easily make cellulose paper satisfy most performance requirements, such as high transparency and good ionic conductivity [23,24]. Nevertheless, most ionically conductive materials are hydrophilic, leading to their overall performance being easily affected by the humidity in the environment [25,26,27]. In response to this challenge, considerable efforts have been dedicated to preparing transparent, hydrophobic and ionically conductive materials such as polymerizable and hydrophobic imidazolium-based ionic liquids (ILs), and a series of multifunctional ionogels that are highly hydrophobic, transparent and ionic conductive have been prepared from these materials [28,29,30]. However, only a limited number of studies have focused on utilizing the hydrophobic and ionically conductive materials to construct hydrophobic cellulose paper with excellent comprehensive performance that would greatly broaden its practical applications.

**Figure 1:**
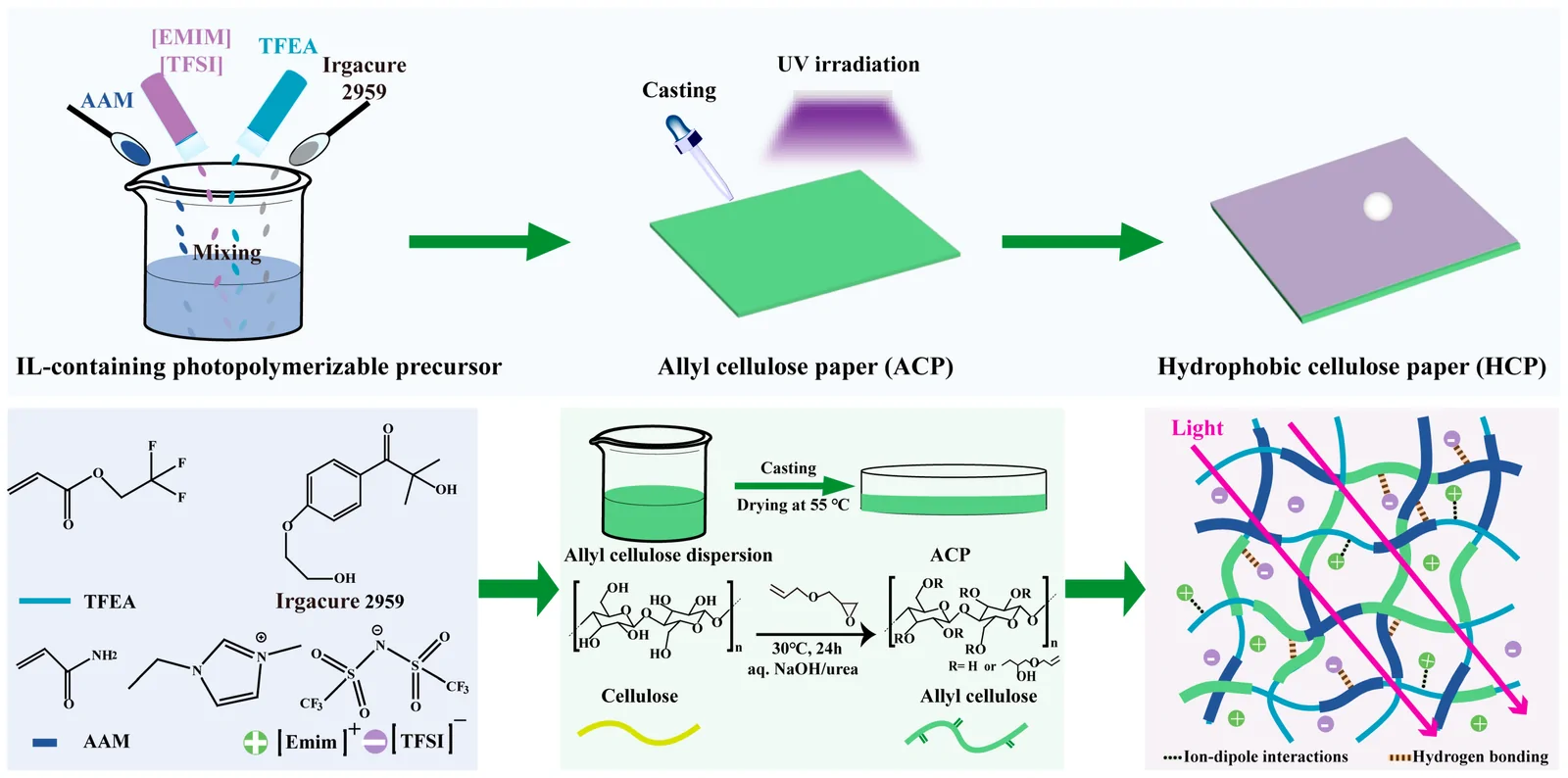
Schematic illustration of the preparation of transparent, conductive and hydrophobic cellulose paper (HCP).

Herein, we tailor the surface hydrophobicity and functionality of cellulose paper via photopolymerization. IL-containing photopolymerizable precursor (Figure S1) was cast onto allyl cellulose paper (ACP) and quickly polymerized via photoinitiated polymerization of 2,2,2-trifluoroethyl acrylate (TFEA), acrylamide (AAM) and allyl cellulose on the surface of ACP to acquire HCP, as shown in Figure 1. The IL-containing photopolymerizable precursor consisted of 1-Ethyl-3-methylimidazolium bis(trifluoromethylsulfonyl)imide ([EMIM][TFSI]), TFEA, AAM, and 2-hydroxy-4′-(2-hydroxyethoxy)-2-methylpropiophenone (irgacure 2959). Transparent fluoropolymers formed by photoinitiated polymerization are known for high stability, inertness, and low surface energy owing to C-F bonds and abundant fluorine atoms, which endow HCP with excellent optical transparency and better surface hydrophobicity [31]. Meanwhile, a large number of noncovalent bonds, including ion-dipole interactions and hydrogen bonding, existed in the system [29], and the photopolymerization of IL-containing photopolymerizable precursor and allyl cellulose on the ACP surface yielded excellent and robust functional surfaces. In addition, the hydrophobic imidazolium-containing IL, [EMIM][TFSI], as an electrolyte salt not only interacts with -CF_3_ groups on fluoropolymers through ion-dipole interactions, improving the stability of performance, but also dominates the ionic conductivity of HCP.

## 2. Results and Discussion

### 2.1. Structure and Optical Properties

**Figure 2:**
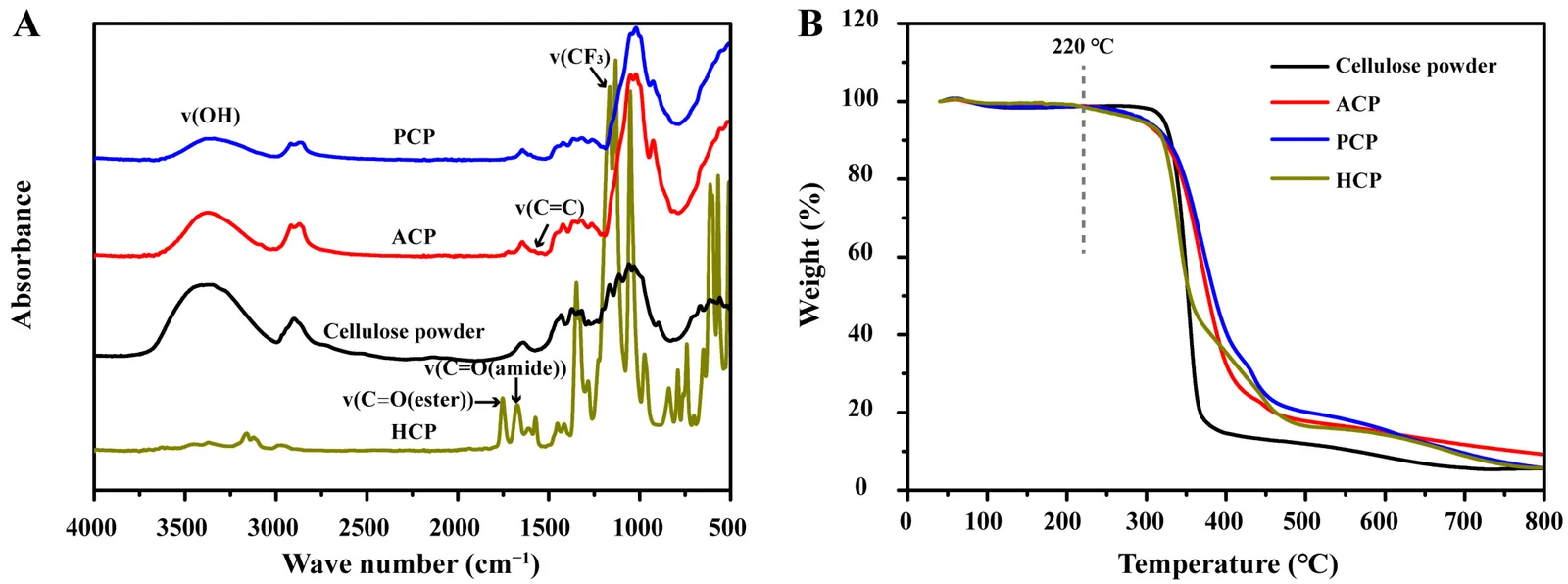
(**A**) ATR-FTIR spectrum and (**B**) TGA curves for cellulose powder, ACP, PCP and HCP.

Attenuated total reflectance-Fourier transform infrared spectroscopy (ATR-FTIR) of cellulose powder, ACP, photocrosslinkable cellulose paper (PCP) and HCP is demonstrated in Figure 2A. In the spectra of ACP, the appearance of a characteristic peak of C=C bonds (1580 cm^−1^) and decreased intensity of O-H bands (3700–3000 cm^−1^) compared with those in the spectra of cellulose powder indicated that allyl glycidyl ether successfully endowed cellulose molecules with double bonds by substituting hydroxyl groups of cellulose chains [32,33]. Noticeably, the peak of C=C bonds almost disappeared in the spectra of PCP, demonstrating the polymerization of allyl cellulose initiated by Irgacure 2959 under UV light. For HCP, there were transparent and hydrophobic fluoropolymers on its surface, and the characteristic peaks of -CF_3_ groups, ester groups, and amide groups of fluoropolymers were observed. The above results implied photopolymerization of IL-containing photopolymerizable precursor and allyl cellulose on the ACP surface. In addition, the thermal stability of various samples was evaluated; the transparent substrates used for fabricated flexible electrical devices need to undergo a processing temperature of at least 150 °C [34]. As shown in Figure 2B, the mass loss of HCP was the lowest in the stage (100–210 °C), which might be due to the excellent surface hydrophobicity of HCP reducing the content of internal bound water and crystalline water [3]. The onset of thermal decomposition of ACP, PCP and HCP was almost the same, at about 220 °C, which was lower than that of cellulose powder. This result might be due to the fact that the glycoside bond (ether bond) generated by the cellulose modification process has reduced thermal stability [17]. In the stage (300–400 °C), the degradation rate (slope of the thermogravimetric analysis (TGA) curve) of PCP was the slowest, which is due to the polymerization of allyl cellulose generating a certain amount of covalent bonds. Noticeably, the degradation rate of HCP was higher than that of PCP and ACP. This result is more likely because the ester bonds of fluoropolymers inside HCP reduce thermal stability [35,36,37].

**Figure 3:**
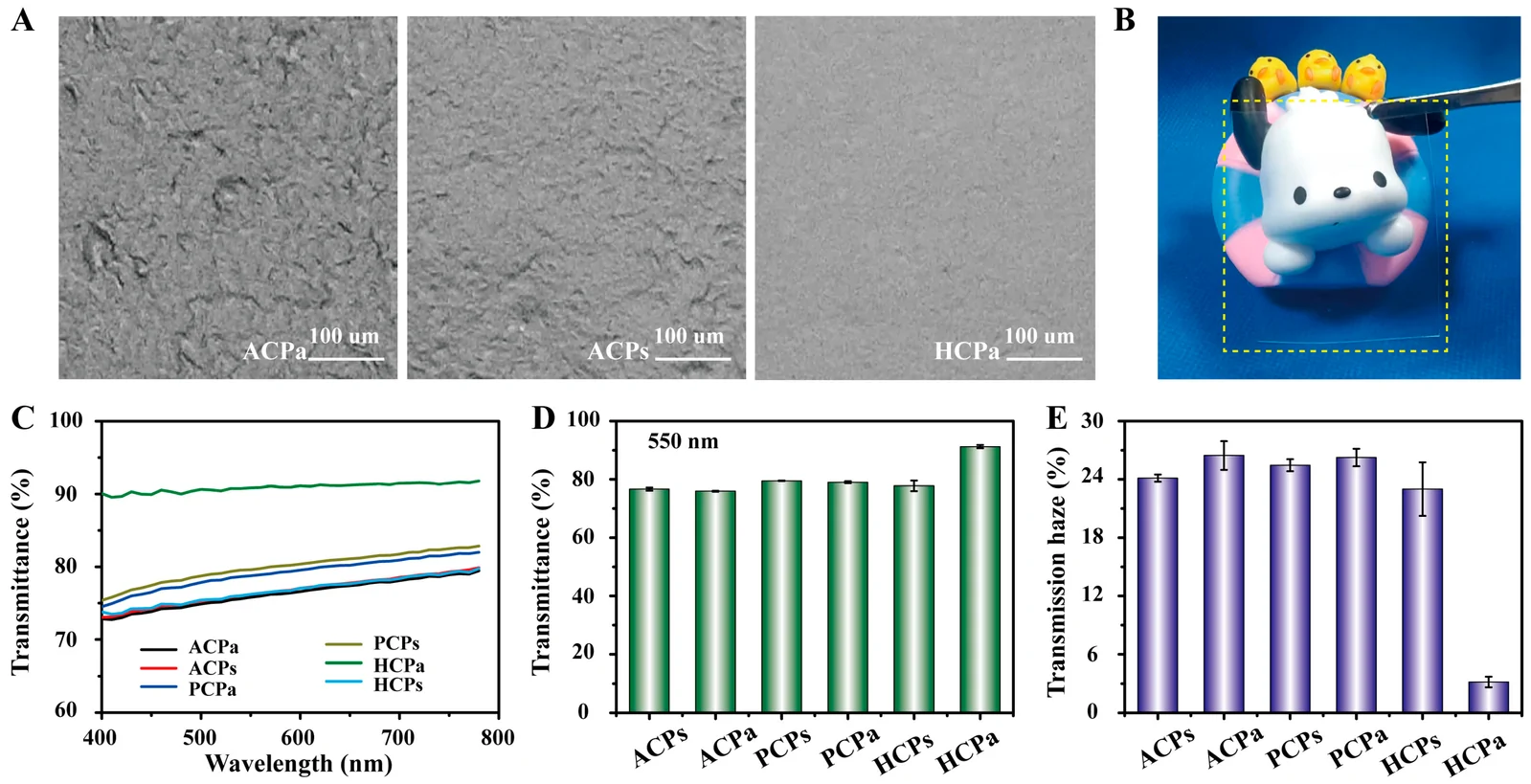
(**A**) Surface morphology of ACPa, ACPs and HCPa. (**B**) Digital photograph of HCPa. This HCPa exhibits a highly transparent property. (**C**) Transmittance spectra of various cellulose paper products. (**D**) Transmittance of various cellulose paper products at 550 nm wavelength. (**E**) Transmission haze of various cellulose paper products.

The morphology of various cellulose papers was investigated (Figure 3A). Obviously, the ACP fabricated by allyl cellulose dispersion cast into a plastic case has micrometer surface roughness. However, the surface of ACPs is denser and smoother than that of ACPa, where s and a represent the substrate side and air side of ACP, respectively. This result might be due to the air side of ACP not constrained by substrate geometry, allowing the allyl cellulose chains to freely aggregate to form high rough surface during drying process [38]. After introduction of a transparent IL-containing photopolymerizable precursor on the air side of ACP and photoinitiated polymerization under UV light, the HCPa displayed a smooth surface that would be conducive to achieving high transparency due to minimizing light scattering [39,40]. The rough surface was replaced by a flat and dense surface of a transparent fluoropolymer layer, and a higher-magnification surface image of HCPa is presented in Figure S2.

The optical properties of HCP are important for optoelectronic devices [41]. Figure 3B exhibits the visual appearance of HCPa. The toy can be clearly observed through the HCPa, indicating that HCPa has high transparency. Transmittance spectra of various cellulose paper products were measured to quantify transparency (Figure 3C). Obviously, the optical transmittance of ACPs was slightly increased compared to that of ACPa. The difference in transparency on both sides of ACP was attributed to the surface of substrate side with lower roughness than that of air side. The transparency on both sides of PCP exhibited a similar pattern to that of ACP. Noticeably, the optical transmittance of PCP was higher than that of ACP, which may be ascribed to photoinitiated polymerization of allyl cellulose leading to a denser surface. Significantly, the optical transmittance of HCPa was dramatically increased in a wide range of wavelengths. This result benefited from the fact that the rougher surface of ACPa was replaced by a fluoropolymer layer, making it smoother and flatter. The transmittance of various cellulose paper products at 550 nm is shown in Figure 3D, and HCPa had a high transparency value (~91%). Meanwhile, HCPa had low transmission haze originating from light scattering (Figure 3E). Therefore, our HCPa shows significant promise in flexible optoelectronic devices that require low transmission haze and high transparency [42].

### 2.2. Hydrophobic and Mechanical Performance

**Figure 4:**
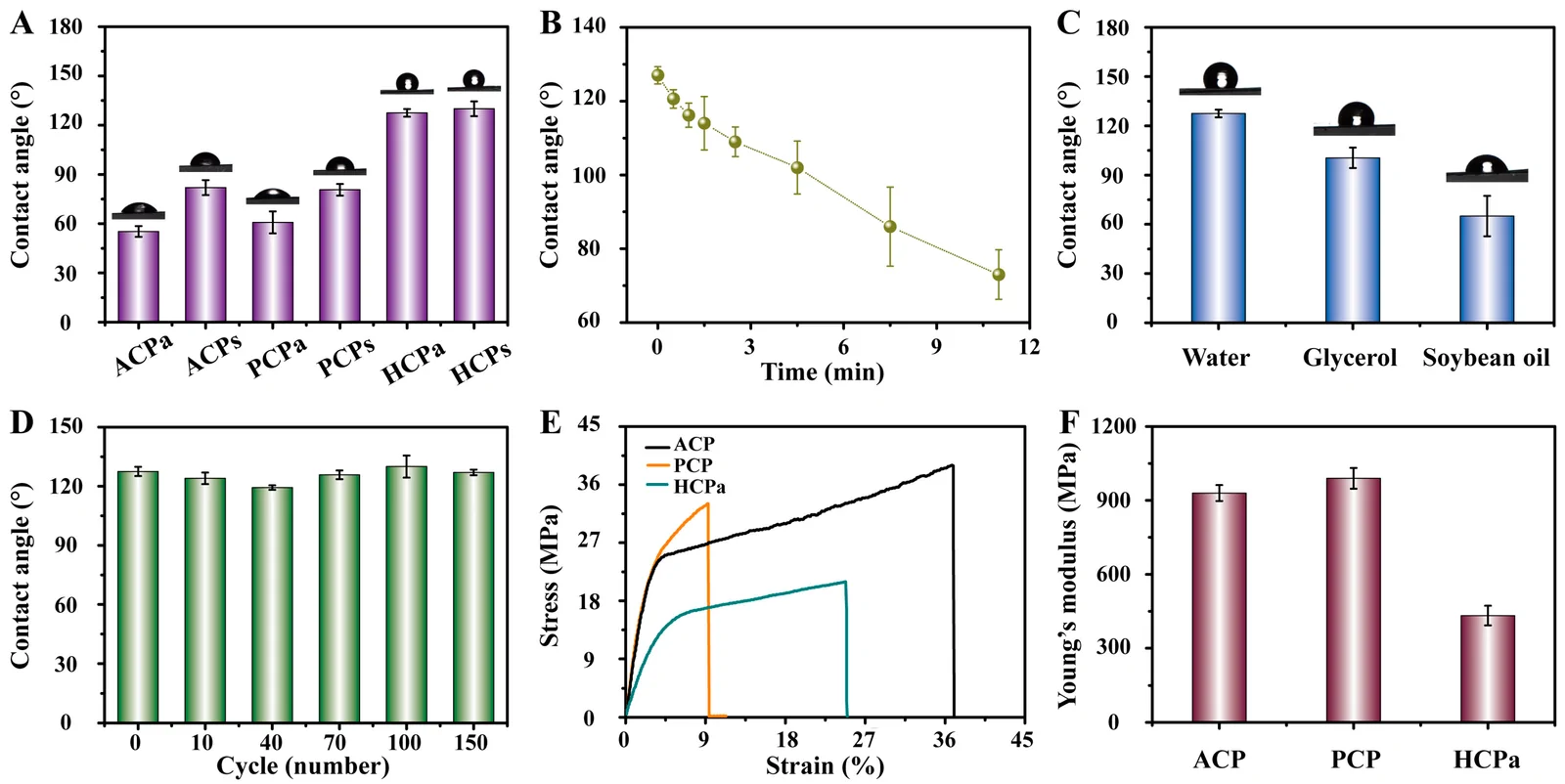
(**A**) Water contact angles of various cellulose paper products. (**B**) Changes in water contact angle of HCPa at different times. (**C**) Contact angles of HCPa for water, glycerol and soybean oil. (**D**) Changes in water contact angle of HCPa under continuous adherence to glass. (**E**) Tensile stress–strain curves and (**F**) Young’s modulus of ACP, PCP and HCPa.

Water contact angles of various cellulose paper products were shown in Figure 4A. ACP and PCP displayed hydrophilic properties (water contact angles <90°), which were attributed to a large number of hydrophilic hydroxyl groups on the cellulose chain, although allyl glycidyl ether had partially replaced the hydroxyl groups [17]. However, the water contact angles of ACPa and PCPa were lower than those of ACPs and PCPs, respectively, which was more likely ascribed to the greater surface roughness making a hydrophilic surface even more hydrophilic [43]. Remarkably, fluoropolymers formed by photoinitiated polymerization possess high stability, inertness and low surface energy owing to C-F bonds and abundant fluorine atoms [31], which endowed HCP with high surface hydrophobic performance. The water contact angles of HCPa and HCPs were almost the same. In addition, HCPa had a smoother surface compared with ACP (Figure 3A). However, a higher-magnification surface image of HCPa (Figure S2) revealed a finely rough surface, which would also contribute to the improvement of hydrophobicity. Thus, the HCPa with excellent optical properties and high surface hydrophobicity was judged to be the favorable cellulose paper for further demonstration.

The HCPa with a high initial water contact angle (~127°) maintained surface hydrophobicity (~102°) after 4.5 min, and its contact angle was still higher than the initial water contact angle of ACPa after 11 min, as shown in Figure 4B. Images of time-dependent water droplets on the HCPa surface (Figure S3) visually displayed the dynamic wetting behavior, which qualitatively characterized the water absorption tendency of the surface. Noticeably, the HCPa surface was not obviously soluble in water, and Xu et al. had demonstrated that fluoropolymers prepared from AAM, TFEA, and [EMIM][TFSI] did not dissolve in water after soaking for 14 days [29]. In addition, HCPa had a high contact angle with hydrophilic glycerol, but a low contact angle with hydrophobic soybean oil (Figure 4C), which may be attributed to the low surface energy of soybean oil [44]. Moreover, the existence of abundant polar groups, such as amide and sulfonyl groups, and trifluoromethyl in fluoropolymers, could provide adhesion properties by noncovalent interactions [29]. As shown in Figure S4, HCPa could adhere to various substrates. Thus, changes in the water contact angle of HCPa under continuous adhesion were investigated (Figure 4D). The results revealed that HCPa still maintained its excellent hydrophobicity after 150 consecutive adhesions to glass, indicating excellent and robust functional surfaces. Mechanical properties were also evaluated as shown in Figure 4E. Tensile strain of PCP was significantly reduced compared to that of ACP, implying that high polymerization density in PCP damaged mechanical performance. Fortunately, the HCPa had moderate tensile strain, which was higher than that of most cellulose-based paper (tensile strain ≤20%) [6,21,45]. Meanwhile, the HCPa had a better Young’s modulus (a measure of stiffness, Figure 4F), which would more easily perceive micro-strains for sensors [46].

### 2.3. Electromechanical and Optoelectronic Device Properties

**Figure 5:**
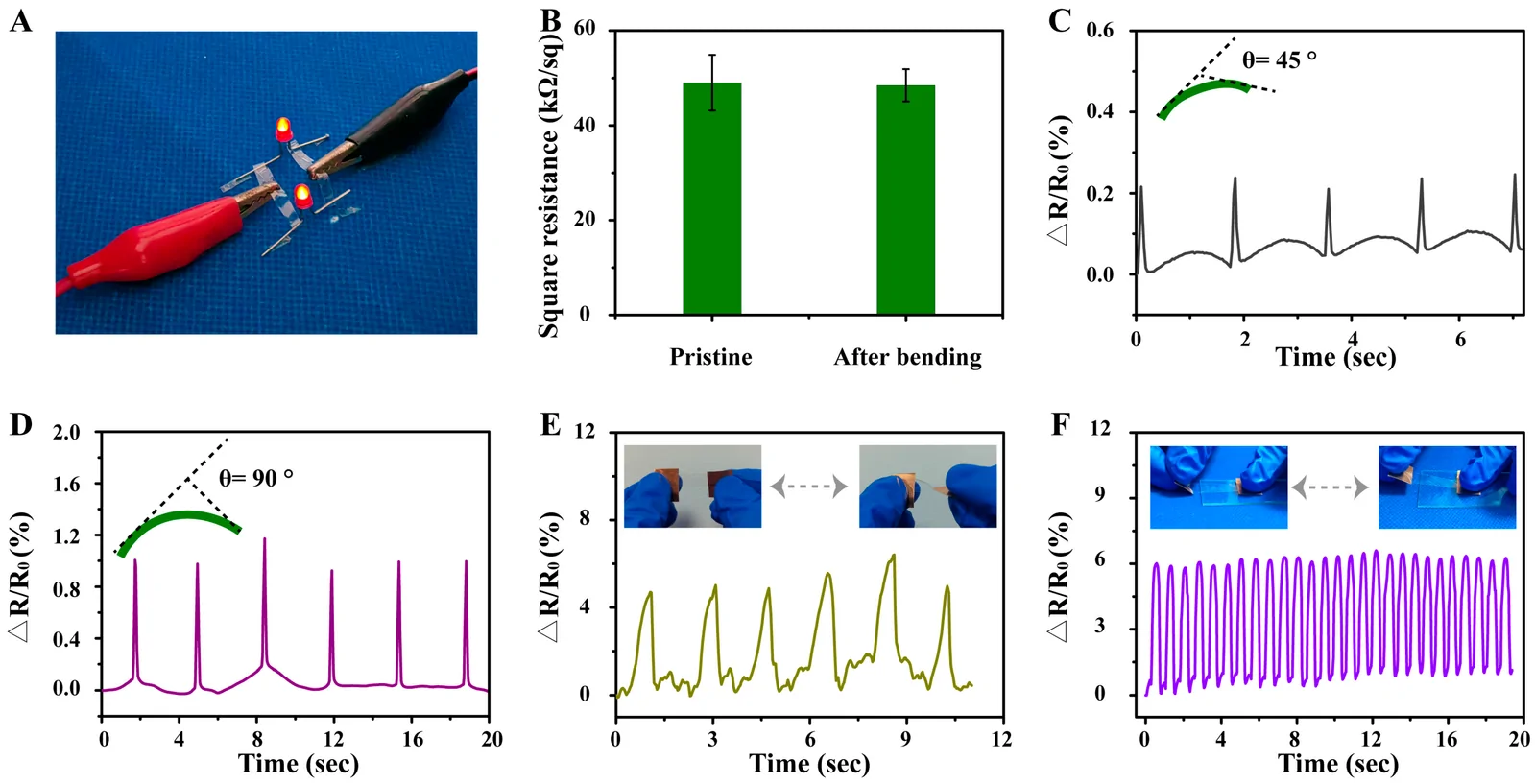
(**A**) Digital photograph of transparent and conductive HCPa in series with an LED. (**B**) Square resistance changes in HCPa after 100 cycles at a bending angle of 150°. Relative resistance changes in HCPa caused by (**C**) a bending angle of 45°, (**D**) a bending angle of 90°, (**E**) twisting, and (**F**) adhering.

The electrolyte salt, [EMIM][TFSI], not only provided ion-dipole interactions and hydrogen bonding for HCP, but also dominated the ionic conductivity of HCP. Figure 5A shows that two LEDs are successfully lit as transparent and conductive HCPa in series with the LED. The square resistance of HCPa was carefully investigated by a four-point probe meter as shown in Figure 5B. The square resistance and fluoropolymer layer thickness of HCPa were ~49 kΩ sq^−1^ and ~0.081 mm, respectively. The corresponding ionic conductivity of HCPa was ~2.52 × 10^−3^ S/cm, which was calculated by δ = 1/(R_s_ × d), where δ is ionic conductivity, R_s_ is square resistance, and d is fluoropolymer layer thickness. The ionic conductivity is higher than that of many other reported ionic sensors, such as ionic conductive nanopaper (~1.3 × 10^−3^ S/cm) [21], cellulose ionic hydrogels (~1.6 × 10^−4^ S/cm) [33], and zwitterionic eutectogel (2.3 × 10^−4^ S/cm) [47]. Noticeably, the square resistance of HCPa hardly changed after 100 cycles at a bending angle of 150°. In addition, resistance changes in conductive HCPa caused by deformation were investigated. As shown in Figure 5C,D, ΔR/R_0_ signals of HCPa immediately increased with external deformation, and exhibited sensitive, uniform and reproducible behavior at the same bending angle. It can also be found that the intensity of ΔR/R_0_ increased as the bending angle increased from 45° to 90°. Similarly, twisting deformation of HCPa could also be monitored by output ΔR/R_0_ signals (Figure 5E). Obviously, the various deformations could be differentiated based on output signal shape and intensity. Furthermore, the electrical performance of HCPa under continuous adhesion was also evaluated (Figure 5F). The output ΔR/R_0_ signals were excellently repeatable and stable after 27 cycles. These observations suggested that the transparent and conductive HCPa had great promise in flexible ionic sensors.

**Figure 6:**
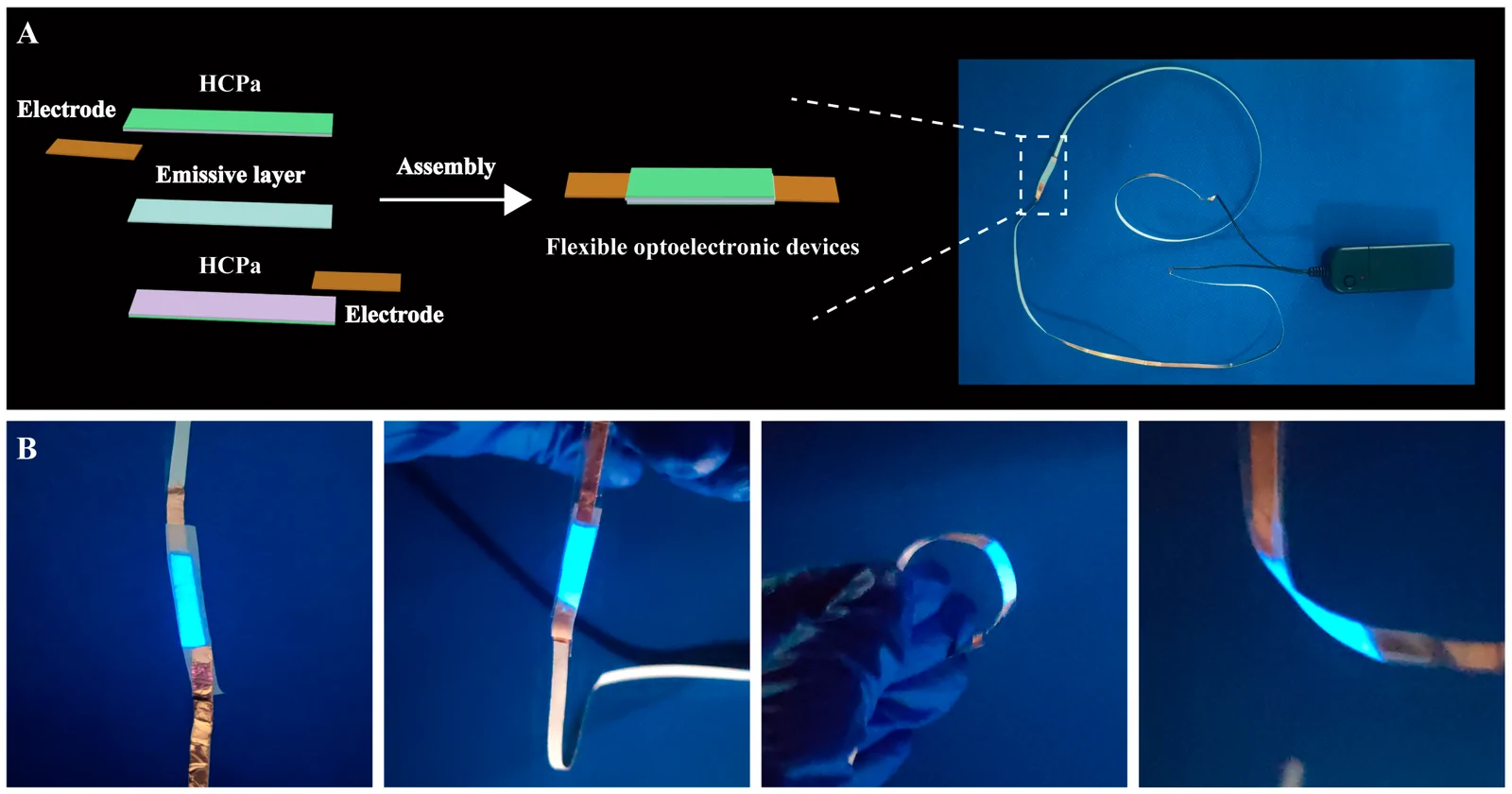
(**A**) Schematic illustration and photograph of HCPa-based flexible optoelectronic devices. (**B**) The optoelectronic devices with horizontal, lifting, bending and twisting, demonstrating integrated and flexible characteristics.

Because of the good optical and electromechanical performances of HCPa, its application, especially as an optoelectronic device, was investigated. Figure 6A shows a schematic illustration and photograph of an HCPa-based flexible optoelectronic device, which is composed of HCPa, an emissive layer and HCPa in sequence. The preparation of the emissive layer was as previously reported [17]. Noticeably, the conductive layer of HCPa needs to be closely touched with emissive layer to ensure that the emissive layer emits light when a high-frequency alternating voltage is applied. The power device obtained from the Shanghai KPT company can convert the direct-current field generated by commercial batteries (3 V) into an alternating-current field with elevated voltage. Figure 6B shows that the optoelectronic devices have been successfully developed. Because HCPa had adhesiveness due to the existence of abundant polar groups in fluoropolymers of HCPa, the optoelectronic devices retained good luminous brightness with horizontal, lifting, bending, and twisting, demonstrating integrated and flexible characteristics.

## 3. Materials and Methods

### 3.1. Chemicals and Materials

Cellulose (powder, 50 μm), sodium hydroxide (≥97%), allyl glycidyl ether (≥99%), [EMIM][TFSI] (≥97%), TFEA (≥98%), AAM (≥99%), irgacure 2959 (≥98%), and glycerol (≥99%) were obtained from Shanghai Aladdin Reagent Co., Ltd. (Shanghai, China). Urea (99%) was obtained from Shanghai Macklin Biochemical Co., Ltd. (Shanghai, China). ZnS:Cu powders (D417) were purchased from Shanghai KPT (Shanghai, China). Silicone elastomer was prepared using SYLGARD 184 (Dow Corning, Midland, MI, USA).

### 3.2. Preparation of Allyl Cellulose

The desired amount of cellulose powder was dispersed into a 7 wt% NaOH/12 wt% urea solution with stirring at −12.5 °C to acquire a 6 wt% cellulose solution. During cellulose dissolution, the NaOH/urea solution will disrupt intra- and intermolecular hydrogen interactions of cellulose, leading to the conversion from cellulose I to cellulose II [33,48]. Allyl glycidyl ether was added dropwise to the stirred cellulose solution until the 8-fold molar ratio of allyl glycidyl ether to anhydroglucose units of cellulose was reached. The mixture reacted at 30 °C for 24 h. Then the mixture was dialyzed in deionized water for 3 days to obtain purified allyl cellulose. The 3 wt% allyl cellulose dispersion with a visually uniform dispersion was prepared by dilution in distilled water with vigorous stirring for 2 h and then stored at 4 °C for future utilization (Figure S5A).

### 3.3. Preparation of IL-Containing Photopolymerizable Precursor

IL-containing photopolymerizable precursor was prepared in a typical procedure [29]. The volume ratio of TFEA to [EMIM][TFSI] was 4:6, and the molar ratio of AAm to TFEA was 40%. The IL-containing photopolymerizable precursor with Irgacure 2959 (0.05 wt%) was strongly stirred at 40 °C for 40 min and stored at this temperature for subsequent use.

### 3.4. Fabrication of HCP

A total of 3 wt% allyl cellulose dispersion was cast into a plastic case, and the liquid level height was 2 mm, which was dried at 55 °C for 24 h to obtain the ACP. The HCP was obtained by casting an IL-containing photopolymerizable precursor onto ACP, and the preparation process was as follows. The ACP was fixed on a glass plate with a release film by scotch tape, and the IL-containing photopolymerizable precursor was dropped onto the ACP, with the release film and glass plate on top in sequence. Subsequently, excess solution was removed with a glass rod. Finally, the assembled sample was exposed to UV light (365 nm, high-strength 40 beads, 80 W, Zhongshan Guba Lighting Co., Ltd., Zhongshan, China) for 10 min to obtain HCP, and the distance of the assembled sample from the irradiation of UV light was ~7.6 cm. The HCPs are designated as HCPa and HCPs, where a and s represent the coating of IL-containing photopolymerizable precursor on the air side and substrate side of ACP, respectively. The PCP was also fabricated. Allyl cellulose dispersion with Irgacure 2959 (0.05 wt%) was cast onto a plastic plate with 2 mm thickness of rubber spacer, and on top of that were a release film and a glass plate in sequence, which was exposed to UV light for 10 min to obtain a gel sheet (Figure S5B). The gel sheet was air-dried for 48 h and then dried in an oven at 55 °C for 24 h, and finally a dry PCP was obtained.

### 3.5. Characterization

ATR-FTIR (Thermo Fisher Scientific Nicolet iS20, Madison, WI, USA) was used for characterization of chemical structures in the range of 4000 cm^−1^ to 500 cm^−1^. TGA was recorded on a PerkinElmer STA 8000 (Waltham, MA, USA) at a heating rate of 10 °C/min, from 40 °C to 800 °C under a nitrogen atmosphere. Surface morphology was observed using a digital microscope (suolan, Zhejiang Rongjiashi Industrial Co., Ltd., Lishui, China). The transmission haze and transparency of samples were measured using a UV-vis spectrometer (CS-820N, Hangzhou Caipu Technology Co., Ltd., Hangzhou, China). Contact angles of 5 uL droplets on samples were recorded on a contact angle meter (SDC-200, Suliang Instrument Technology Co., Ltd., Suzhou, China). The tensile tests were performed on a tensile machine (DR-506QS, Dongguan Dongri Instrument Co., Ltd., Dongguan, China) at a tensile speed of 5 mm/min. A four-point probe meter (RTS-9, Guangzhou Four-Point Probe Technology Co., Ltd., Guangzhou, China) was used for measurement of square resistance. The real-time resistance characteristics were tested by digital multimeter (Keithley DMM7510, Tektronix Corporation, Solon, OH, USA).

## 4. Conclusions

In summary, construction of highly transparent and ionically conductive HCP is realized via photopolymerization of IL-containing photopolymerizable precursor and allyl cellulose on the surface of ACP. This new strategy endows HCP with excellent optical transparency (>90% in a wide wavelength range), better surface hydrophobicity (water contact angle ~127°) and robust functional surfaces. In addition, the HCP also exhibits high thermostability (onset thermal decomposition ~220 °C), as well as reliable, sensitive and reproducible responses to external stimuli. Significantly, the HCP with good optical and electromechanical performances has successfully been used for the construction of integrated and flexible optoelectronic devices. Subsequent research will be needed to explore the device stability and performance to comprehensively evaluate the practical prospects of conductive HCP. The present work gives a new avenue for tailoring the surface hydrophobicity and functionality of cellulose paper that has great promise in the flexible electronics field.

## Data Availability

The data that support the findings of this study are available upon reasonable request from the authors.

## Supplementary Materials

The following supporting information can be downloaded at: https://www.mdpi.com/article/10.3390/molecules31203711/s1. Figure S1: (A) Transparent ionic liquid-containing photopolymerizable precursor (IL-containing photopolymerizable precursor). (B) Transparent and stretchable ionogels that were prepared via photoinitiated polymerization of IL-containing photopolymerizable precursor. Figure S2: Higher-magnification surface image of hydrophobic cellulose paper (HCPa). Figure S3: Images of time-dependent water droplets on HCPa surface. Figure S4: Photographs of HCPa adhering to various substrates. Figure S5: Photographs of (A) allyl cellulose suspension and (B) gel sheet obtained through the photocrosslinking of allyl cellulose.

## Abbreviations

The following abbreviations are used in this manuscript:

HCP	Hydrophobic cellulose paper
IL	Ionic liquid
ACP	Allyl cellulose paper
[EMIM][TFSI]	1-Ethyl-3-methylimidazolium bis (trifluoromethylsulfonyl)imide
TFEA	2,2,2-trifluoroethyl acrylate
AAM	Acrylamide
Irgacure 2959	2-hydroxy-4′-(2-hydroxyethoxy)-2-methylpropiophenone
PCP	Photocrosslinkable cellulose paper
ATR-FTIR	Attenuated total reflectance-Fourier transform infrared spectroscopy
TGA	Thermogravimetric analysis
