# Supplementary material for: Transparent, Conductive and Hydrophobic Cellulose Paper Fabricated by Photopolymerization
Source: Molecules. 2026 Oct 16;31(20):3711. doi: 10.3390/molecules31203711 (PMC13707420; doi:10.3390/molecules31203711)
Supplement: Supplementary file 1 [file molecules-31-03711-s001.zip › molecules-4599827-supplementary.pdf]

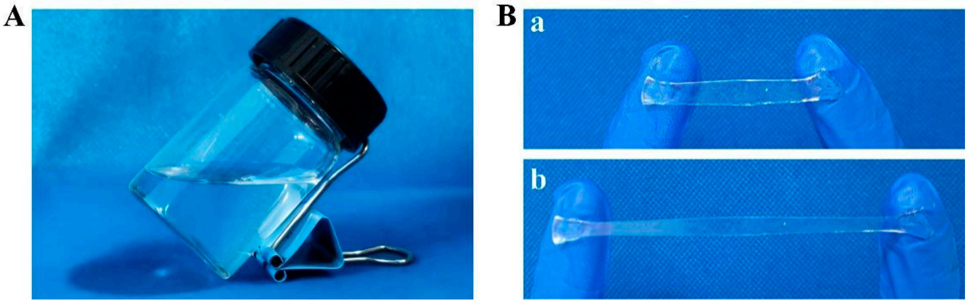

Figure S1. (A) Transparent ionic liquid-containing photopolymerizable precursor (IL-containing photopolymerizable precursor). (B) Transparent and stretchable ionogels that were prepared via photoinitiated polymerization of IL-containing photopolymerizable precursor.

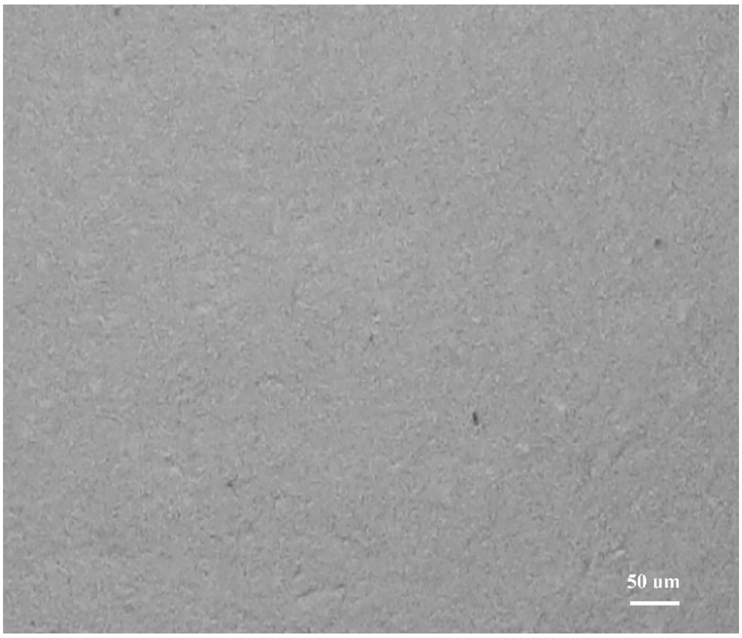

Figure S2. Higher-magnification surface image of hydrophobic cellulose paper (HCPa).

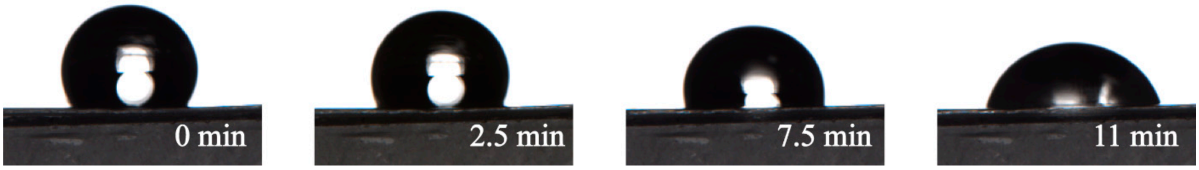

Figure S3. Images of time-dependent water droplet on HCPa surface.

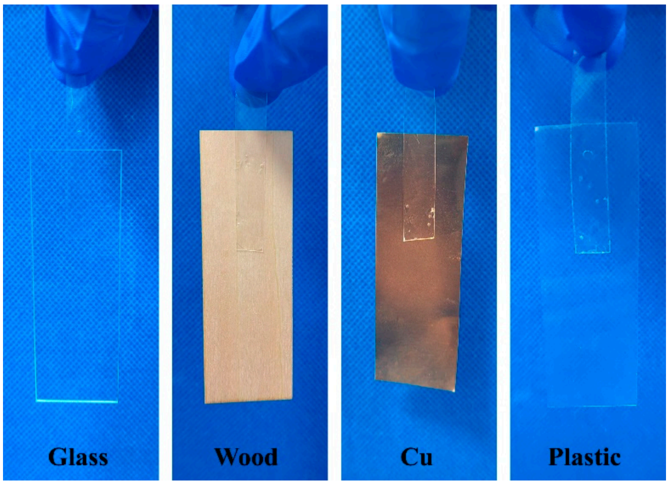

Figure S4. Photographs of HCPa adhering to various substrates.

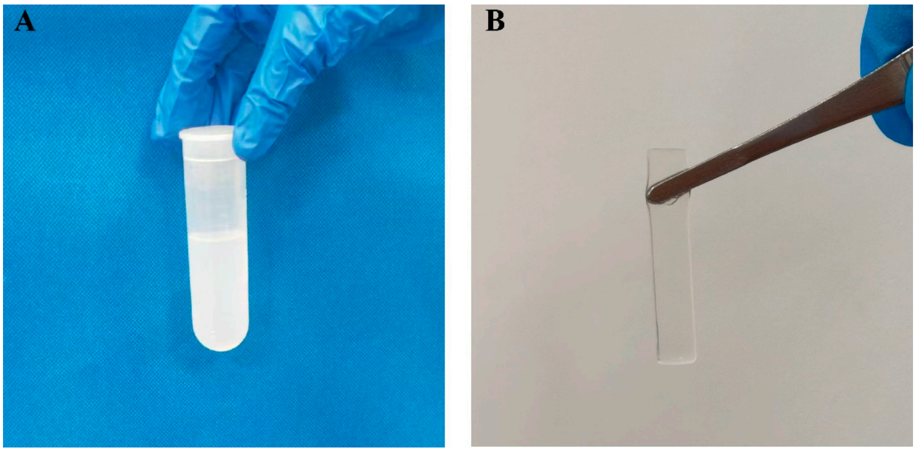

Figure S5. Photographs of (A) allyl cellulose suspension and (B) gel sheet obtained through the photocrosslinking of allyl cellulose.
